# Streptococcal infection and autoimmune diseases

**DOI:** 10.3389/fimmu.2024.1361123

**Published:** 2024-02-23

**Authors:** Ayaka Ohashi, Masanori A. Murayama, Yoshishige Miyabe, Kazuo Yudoh, Chie Miyabe

**Affiliations:** ^1^ Department of Immunology and Parasitology, St. Marianna University School of Medicine, Kawasaki, Japan; ^2^ Department of Animal Models for Human Diseases, Institute of Biomedical Science, Kansai Medical University, Osaka, Japan; ^3^ Department of Frontier Medicine, Institute of Medical Science, St. Marianna University School of Medicine, Kawasaki, Japan

**Keywords:** *Streptococcus*, infection, autoimmune diseases, nephritis-associated plasmin receptor (NAPlr), molecular mimicry

## Abstract

Excessive activation of immune cells by environmental factors, such as infection or individual genetic risk, causes various autoimmune diseases. *Streptococcus* species are gram-positive bacteria that colonize the nasopharynx, respiratory tract, gastrointestinal tract, genitourinary tract, and skin. Group A *Streptococcus* (GAS) species cause various symptoms, ranging from mild infections, such as tonsillitis and pharyngitis, to serious infections, such as necrotizing fasciitis and streptococcal toxic shock syndrome. The contribution of GAS infections to several autoimmune diseases, including acute rheumatic fever, vasculitis, and neuropsychiatric disorders, has been studied. In this review, we focus on the association between streptococcal infections and autoimmune diseases, and discuss current research on the mechanisms underlying the initiation and progression of autoimmune diseases.

## 1 Introduction

Streptococci are anaerobic, gram-positive organisms that constitute a heterogeneous group of bacteria that include more than 100 species (1). Streptococci colonize mucosal tissues in the nasopharynx, respiratory tract, gastrointestinal tract, genitourinary tract, and skin (2). Some of these bacteria are harmless to humans; however, some pathogenic species can cause severe infections (3). Streptococcal infections were first reported in 4^th^ century BC, when Hippocrates described the disease erysipelas; however, major advancements were made through microscopic observation and the subsequent description of their forms by Anton van Leeuwenhoek (4). *Streptococcus* was first isolated from the uteri and blood of women with puerperal fever by Louis Pasteur in 1879 and was shown to be the etiological microbe responsible for a condition that caused the highest mortality rates among women and newborns (4). Streptococci were classified based on the Lancefield scheme, which groups streptococcal strains according to the carbohydrate composition of cell wall antigens, such as polysaccharides (groups A, B, C, E, F, and G), teichoic acids (groups D and N), and lipoteichoic acid (group H) (2).

*Streptococcus pyogenes*, also known as Group A *Streptococcus* (GAS), causes mild infections, such as respiratory tract infections, including tonsillitis and pharyngitis, and rarely causes serious infections such as necrotizing fasciitis and streptococcal toxic shock syndrome (STSS), which shows similar symptoms to toxic shock syndrome (TSS) caused by *Staphylococcus aureus* (5). GAS is also known to colonize the skin, causing superficial infections such as pyoderma and impetigo (5, 6). The prevalence of GAS among healthy children with no signs or symptoms of pharyngitis is 12% (7). GAS is considered the most clinically important Streptococcal group due to the high number of cases and clinically important postinfectious sequelae, such as post-streptococcal glomerulonephritis and acute rheumatic fever (8).

**Figure 1:**
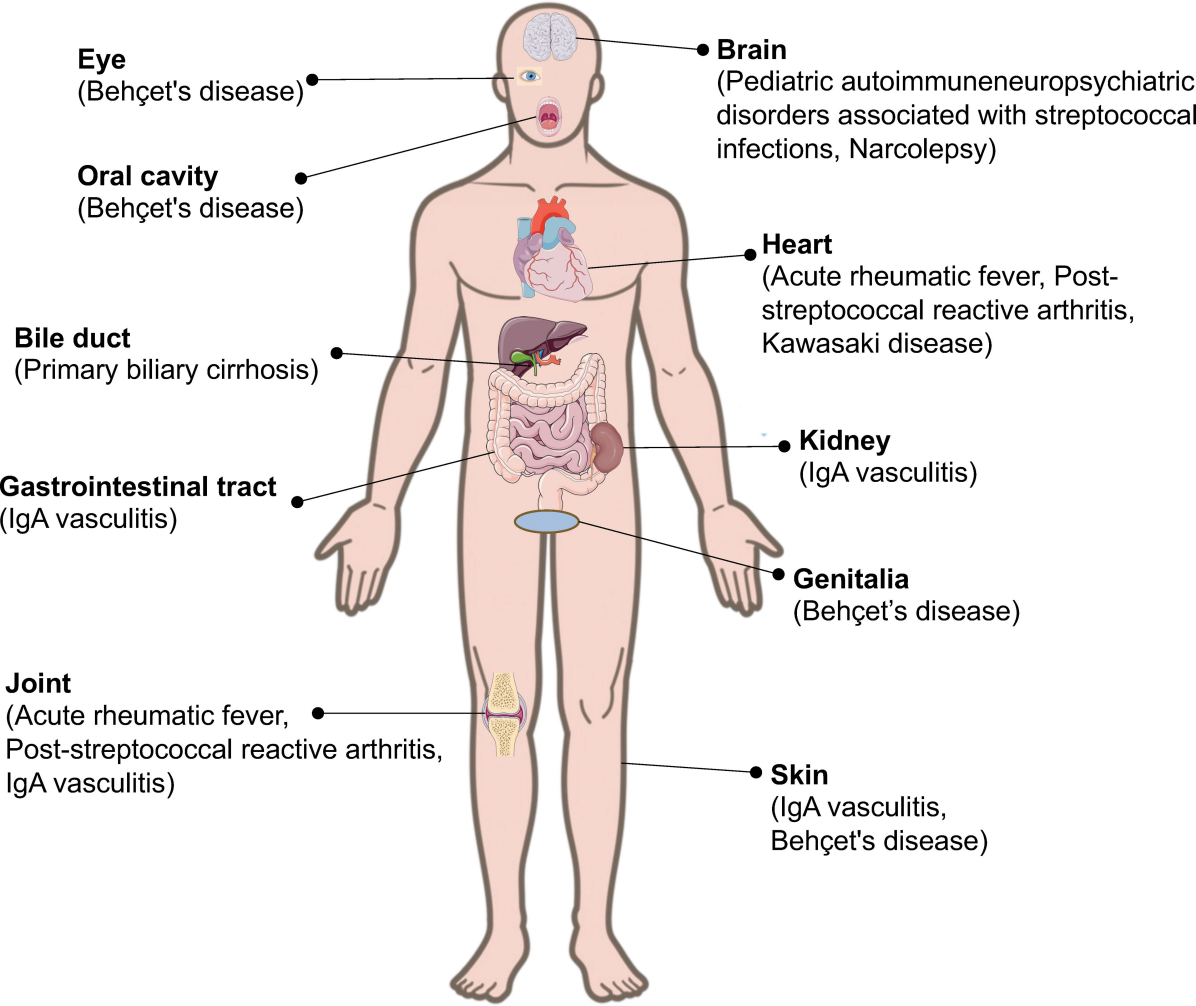
Streptococcal infection and its related autoimmune diseases. Streptococcal infection can induce autoimmune diseases in different parts of the body.

Autoimmune diseases are heterogeneous disorders characterized by autoreactive immune responses that result in immune-mediated organ damage (9). Autoimmune diseases are heterogeneous, with a wide range of clinical presentations and courses. Autoimmune diseases can affect virtually every organ in the body, including the heart, blood vessels, skin, joints, kidneys, and central nervous system (10, 11). The association between autoimmune diseases and microbes has been widely reported (12–14). For example, immunoglobulin (Ig)A vasculitis (IgAV) is often triggered by a prior infection, such as an upper respiratory tract infection caused by various microorganisms, including *Streptococcus, S. aureus*, and varicella-zoster virus (13). Although *Streptococcus* is associated with a variety of autoimmune rheumatic diseases (**Figure 1**), it has not received as much attention as other bacteria, such as *S. aureus.*

In this review, we provide an overview of the association between streptococcal infections and autoimmune diseases, thereby contributing to our understanding of the pathogenesis of autoimmune diseases.

## 2 Arthritis

### 2.1 Acute rheumatic fever

Acute rheumatic fever (ARF) is an autoimmune response to GAS infection. ARF may develop approximately two–four weeks after pharyngeal infection with GAS (15), and is characterized by inflammation of the joints (35–66%) and heart (50–70%), typically manifesting as polyarthritis and valvular regurgitation (16, 17). These symptoms are followed by chorea (10–30%), subcutaneous nodules (0–10%), and erythema marginatum (<6%), which are less common but highly specific manifestations of ARF (17). In 1944, the major and minor manifestations of ARF were defined in the Jones criteria to facilitate accurate ARF diagnoses (18). In most cases, the manifestations of acute rheumatic fever resolve without sequelae. However, valvular lesions can persist in some cases, leading to chronic rheumatic heart disease (RHD). Although ARF itself is not usually life-threatening, cardiac involvement can result in heart failure, stroke, or death (16). It is reported that ARF develops in 0.3-3% of people with untreated or ineffectively treated GAS infections (typically children between the ages of 5 and 14 years) (19). It is also estimated that 33 million cases of RHD are responsible for over 275,000 deaths each year (15). Although the pathogenesis of ARF remains unclear, it is considered to be the result of an autoimmune response to pharyngeal infection with GAS in genetically predisposed individuals, which is mediated by molecular mimicry (16). The cell wall of GAS is composed of N-acetylβD-glucosamine linked to a polymeric rhamnose backbone (20). GAS contains M, T, and R surface proteins, as well as lipoteichoic acid, which are involved in bacterial adherence to epithelial cells (20). The M protein is the most important antigenic structure in bacteria and shares structural homology with alpha helical coiled-coil human proteins, such as cardiac myosin, tropomyosin, keratin, laminin, vimentin, and valvular proteins. Mice immunized with streptococcal cell wall and membrane components produce monoclonal antibodies that recognize the human antigens of cardiac structures (21). M proteins can be divided into groups with a preponderance of various symptoms (22). The accumulated data indicate that the biological characteristics of GAS, which has an affinity for the throat, are related to the number of M protein types 1, 3, 5, 6, 14, 18, 19, 24, and 29, the particular *emm* gene patterns, and share epitopes with human heart tissue (23). Although a potential role of Superantigen (SAg) in the development of RHD has previously been studied, experiments designed to test this question remain scarce and are conflicting (15).

### 2.2 Post-streptococcal reactive arthritis

Post-streptococcal reactive arthritis (PSRA) is another type of arthritis associated with streptococcal infections in patients who do not fulfill the Jones criteria for the diagnosis of ARF (24). After acute streptococcal pharyngitis, prolonged symptoms of arthritis and multisystem manifestations that do not fulfill the Jones criteria for the diagnosis of ARF indicate PSRA (25). In ARF, arthritis usually develops 10-28 days after GAS-mediated pharyngitis, while in PSRA, arthritis appears after a shorter “incubation” period; approximately 7-10 days after infection (26). The presentation could be similar to that of acute septic arthritis with a sudden onset of fever and severe joint pain (27). Patterns of joint involvement may be monoarticular, polyarticular, migratory, additive, or chronic. The most commonly involved joints are the large joints, such as the knees and ankles, rather than the small joints of the hands and wrists (25). Arthritis is usually non-destructive and self-limiting; however, symptoms may last for months. The response of patients with PSRA to aspirin has been found to be less effective when compared to those with ARF. Whether PSRA is distinct from ARF has not yet been fully addressed. However, since there are substantial clinical, immunological, and genetic differences between PSRA and ARF, PSRA is considered to be a distinct entity (24, 26). There is limited literature on the pathogenesis of PSRA and the hypothesis has not been fully validated yet, but cross reactivity between virulent molecules of GAS and articular proteins such as keratin, vimentin, and laminin has been extrapolated (28).

## 3 Vascular disease

### 3.1 IgA vasculitis

IgAV, previously referred to as Henoch-Schönlein purpura, is a type of vasculitis characterized by IgA-dominant immune deposits that affect small vessels, including the skin, kidney, gastrointestinal tract, and joints (29). Although the disease mechanism has not yet been elucidated, previous studies have identified various infections as a potential trigger for this disease (30). Several bacterial and viral species are associated with the pathogenesis of IgAV. Representative causative pathogens include *Streptococcus*, *S. aureus*, *Helicobacter pylori*, varicella-zoster virus, hepatitis virus, parvovirus, human immunodeficiency virus, cytomegalovirus, and *Clostridium difficile* (31). Among them, the most frequently isolated microbe is β-hemolytic *Streptococcus* (32). Nephritis-associated plasmin receptor (NAPlr) has been isolated from GAS as a nephritogenic protein in post-streptococcal acute glomerulonephritis (PSAGN) and was shown to be identical to streptococcal glyceraldehyde-3-phosphate dehydrogenase (GAPDH) (33). NAPlr deposition has been detected in the glomeruli of early phase PSAGN, and its distribution is identical to that of plasmin activity, suggesting that NAPlr causes glomerular damage by trapping plasmin and maintaining plasmin activity (33). NAPlr immunoreactivity has been observed in the glomeruli of patients with IgAV with nephritis (34). Recently, we demonstrated that NAPlr was positive in 75.0% of skin tissues derived from patients with IgAV (14). NAPlr immunoreactivity was detected around small dermal vessels, and its distribution was similar to the area where plasmin activity was observed, suggesting that NAPlr-positive perivascular deposits may trigger neutrophil infiltration in IgAV (14). Similar to several other vascular disorders, anti-endothelial cell antibodies (AECA) have been detected in patients with IgAV (35). In IgAV, AECA are exclusively the IgA isotype and directed toward β2-glycoprotein I on the endothelial cell surface (36). IgA AECA may bind to the endothelial cells and enhance IL-8 production via mitogen-activated protein kinase/extracellular regulated kinase pathway and ICAM-1 expression, which is responsible for the perivascular neutrophil infiltration and leukocytoclastic vasculitis in IgAV (37).

### 3.2 Kawasaki disease

Kawasaki disease (KD) is the most common form of systemic vasculitis affecting children and infants (38). KD initially presents with high fever, mucocutaneous inflammation, and cervical lymphadenopathy, followed by inflammation of the coronary arteries and other cardiovascular structures (39). KD is the leading cause of acquired heart disease in children in developed countries (40). Scarlet fever caused by GAS presents with symptoms similar to those of KD, including exanthema, enanthema, and cervical lymphadenopathy. However, purulent tonsillitis and the absence of conjunctivitis in scarlet fever can help differentiate between the two diseases (38). Infectious agents are suspected to trigger KD due to the clinical symptoms and seasonal peaks (41). Several microorganisms, such as Streptococci, *S. aureus*, *Propionibacterium acne*s, and *Yersinia pseudotuberculosis*, have been reported to cause KD; however, their causal relationships are yet to be confirmed (42). Metagenomic approaches have revealed the presence of *Streptococcus* spp. in the lymph nodes and guts of patients with KD (43, 44). SAgs are the powerful T cell stimulators that simultaneously activate the major histocompatibility complex class II (MHC II) molecules and specific Vβ segments of T cell receptors, leading to the activation of various immune cells (45). In Kawasaki disease, *S. aureus* strains expressing various SAg have been isolated, in particular the toxic shock syndrome toxin-1 (TSST-1), and analysis of the Vβ repertoire on the T cell receptor of circulating T cells indicated T cell expansion compatible with SA-driven T cell proliferation (46). The SAg genes of GAS, such as *SPE-A*, *SPE-G*, and *SPE-J*, were also detected more frequently in the stools of children with KD than in those without KD, suggesting that GAS is harbored in the upper airways or gastrointestinal tracts of patients with KD (47). The SAgs of GAS also function as highly potent activators of T lymphocytes and are recognized as causative molecules of the STSS (48). Of interest, some of the streptococcal and staphylococcal SAgs have amino acid sequence homologies, indicating that they have all evolved from a single primordial superantigen (49, 50). For example, TSST-1 of *S. aureus* and SMEZ of GAS have similar amino acid sequences, and cause TSS and STSS, respectively (50). Although less is known about the relationship between SAgs and autoimmune diseases including KD, it is possible that the variation of the structure of SAgs could result in differences in the affinity toward MHC II or the T cell receptors, which may determine the distinct clinical manifestations as a consequence.

## 4 Neuroimmune diseases

### 4.1 Pediatric autoimmune neuropsychiatric disorders associated with streptococcal infections(PANDAS)

Pediatric autoimmune neuropsychiatric disorders associated with streptococcal infections (PANDAS) are acute, sudden onset, obsessive-compulsive disorders accompanied by a previous streptococcal infection (51). PANDAS is characterized by impairment in the basal ganglia, which are responsible for switching between motor and mental behaviors (52). The clinical course of PANDAS is characterized by relapsing-remitting symptoms with significant psychiatric comorbidities accompanying the exacerbations, such as emotional lability, separation anxiety, nighttime fears and bedtime rituals, cognitive deficits, oppositional behaviors, and motoric hyperactivity (53). A major virulence factor of GAS, namely M protein types 5, 6, and 19, has been detected in the human brain using immunofluorescence staining (54). It has been hypothesized that M proteins trigger the production of antineuronal antibodies, such as antibodies against both D1 and D2 receptors, tubulin and lysoganglioside (55–58). These antineuronal autoantibodies are elevated in the serum and cerebrospinal fluid of patients with PANDAS. Antineuronal antibodies are considered to bind to the receptors on the surface of neuronal cells and trigger the signaling cascade of calcium calmodulin-dependent protein kinase II, tyrosine hydroxylase, which may lead to excess dopamine and the manifestations of PANDAS (59). Studies on mice infected intranasally with GAS have demonstrated the importance of GAS infections and its role in opening the blood-brain barrier, which must be broken to allow IgG to penetrate the brain (21).

### 4.2 Narcolepsy

Narcolepsy is a sleep disorder characterized by excessive daytime sleepiness, cataplexy, sleep paralysis, hallucinations, and fragmented nocturnal sleep. It usually occurs during adolescence or young adulthood (60). Narcolepsy is strongly associated with the presence of the *HLA-DQB1n0602* allele, a variant of the *HLA-DQB1* gene. An inciting event, such as infection or vaccination, triggers the destruction of hypocretin-secreting neurons in the hypothalamus, leading to symptoms of narcolepsy (61). A high prevalence of streptococcal throat infections prior to onset has been reported, particularly in prepubertal and peripubertal children (62). It has been hypothesized that *Streptococcus* has a tropism for hypocretin-secreting neutrons, and its infections can increase the risk of narcolepsy through the activation of general immunity or increased hemato-encephalic barrier permeability to T cells (63). However, despite extensive research, auto-antibodies directed toward hypocretin-secreting neurons have not been proved (64).

## 5 Other autoimmune/inflammatory diseases

### 5.1 Primary biliary cirrhosis

Primary biliary cirrhosis (PBC) is a chronic liver disease that predominantly affects middle-aged women and is characterized by the destruction of small intrahepatic bile ducts, portal inflammation, and progressive scarring (65). The presence of elevated antimitochondrial antibody titers facilitates its recognition and the early detection of associated autoimmune diseases such as Hashimoto’s thyroiditis (66). Several bacterial strains have been proposed to cause PBC via molecular mimicry (67). A number of microorganisms have been identified in the tissue or body fluids of patients with PBC, including *Escherichia coli*, retroviruses, Epstein-Barr virus, *Streptococcus intermedius*, *S. aureus*, and *P. acnes* (66, 67). Patients with PBC possess higher IgM and IgA class anti-lipoteichoic acid serum titers, which are major components of the gram-positive streptococcal cell wall that mediate the attachment of bacteria to host tissues (68). IgM class *S. intermedius* titers are significantly higher in the serum of patients with PBC, and bacterial histone-like DNA-binding proteins may be involved in the of PBC (69). Metagenomic analysis of gut microbes in patients with PBC revealed that *Streptococcus* sp. increased nearly five-fold in mean relative abundance, and may serve as a biomarker to distinguish between patients with PBC and healthy individuals (70). The most widely studied mechanism explaining these observations is the molecular mimicry between bacterial antigens and human epitopes. Another hypothesis is that when bacteria enter the mucosa, bacterial lipoic acid residues may be modified by xenobiotics to form immunoreactive products (67). As most xenobiotics are metabolized in the liver, the potential for liver-specific protein alterations would increase.

### 5.2 Behçet’s disease

Behçet’s Disease (BD) is a systemic inflammatory syndrome characterized by relapsing episodes of oral aphthous ulcers, genital ulcers, skin lesions, ocular lesions, and other manifestations including vascular, gastrointestinal, or neurological involvement (71). Histocompatibility leukocyte antigen (HLA)-B*51 is the strongest genetic risk factor of BD (72). Infectious agents (e.g., *Streptococcus sanguinis*, herpes simplex virus, and mycobacteria) have long been proposed as triggering factors for BD pathogenesis (72). An old study reported an abnormal abundance of *S. sanguinis* in the ulcers of patients with BD (73). Another study demonstrated that patients with BD exhibited a positive skin prick specific to intraoral Streptococci (74). Antigens from *S. sanguinis* are suspected to have high homology with human heat-shock proteins (HSPs) and cross-reactivity leads to an immune response (75). HSPs, which are mainly expressed in mitochondria, are conserved in microorganisms and mammals, and serve as antigens for T cell activation (73). Antibodies against *S. sanguinis* and GAS have been detected more frequently in patients with BD than in controls (76). Hyperactivity against microorganisms and increased serum levels of alarmins in patients with genetic risk factors are considered to cause constitutive NF-kB hyperactivation and neutrophil activation, which are prominent features of BD (72).

## 6 Future directions/conclusions

The ability of leukocytes to enter tissues in response to immune stimuli is a central feature of host defense (13). However, the excessive activation of leukocytes aggravates inflammation, leading to various autoimmune diseases. GAS can cause diverse skin and respiratory tract symptoms, and occasionally triggers autoimmune sequelae that persist after the infection resolves due to specific features, such as the direct deposition of streptococcal antigens, molecular mimicry, SAgs, modification of bacterial antigens by xenobiotics, or tropism for a specific organ (**Table 1**). The mechanism by which Streptococci induce persistent autoimmunity has been partially elucidated; however, some questions remain. The common streptococcal antigens that induce autoimmunity and the precise mechanisms by which these antigens promote immune cell attack in specific organs have not been identified. It is also difficult to verify the cause-and-effect relationship between microbes and diseases due to the continuous interaction between these two factors and the lack of appropriate animal models. Further studies in human and animal models are necessary to verify the role of Streptococci in autoimmune diseases, and to identify novel therapeutic and preventive strategies.

**Table 1:** Summarized findings of the association of Streptococcal infection with autoimmune diseases.

Disease	Antigens (Human/Streptococcus)	Immune cells	Mechanism	References
Acute rheumatic fever	cardiac myosin, tropomyosin, keratin, laminin, vimentin	T cells, B cells	molecular mimicry	(21, 23)
IgA vasculitis	NAPlr β2-glycoprotein I	Neutrophils T cells, B cells	deposition of the bacterial antigen molecular mimicry	(14, 34) (35–37)
Kawasaki disease	SPE-A, SPE-G, SPE-J	T cells	superantigens	(46, 47)
PANDAS	dopamine D1 and D2 receptors lysogangliosid, tubulin	T cells, B cells	molecular mimicry	(53–56)
Narcolepsy	hypocretin-secreting neurons?	T cells, B cells?	molecular mimicry?	(61, 62)
Primary biliary cirrhosis	mitochondrial antigens, histone	T cells, B cells	molecular mimicry xenobiotics	(64–67) (65)
Behçet's disease	human heat-shock proteins	T cells, B cells	molecular mimicry	(71, 73)

NAPlr, nephritis-associated plasmin receptor; PANDAS, Pediatric autoimmune neuropsychiatric disorders associated with streptococcal infections.

## Author contributions

AO: Visualization, Writing – original draft, Writing – review & editing. MM: Writing – review & editing. YM: Supervision, Writing – review & editing. KY: Supervision, Writing – review & editing. CM: Writing – original draft, Writing – review & editing, Conceptualization.
